# Preliminary study on heavy metal concentrations of Anatolian Khramulya, *Capoeta tinca* (Heckel, 1843) from Çamlıgöze Dam Lake, Sivas, Turkey

**DOI:** 10.1186/2052-336X-11-7

**Published:** 2013-06-13

**Authors:** Seher Dirican, Süleyman Çilek, Hakan Çiftçi, Mutluhan Bıyıkoğlu, Servet Karaçınar, Ahmet Yokuş

**Affiliations:** 1 Department of Fisheries, Suşehri Vocational Training School, Cumhuriyet University, Suşehri, Sivas, Turkey; 2 Department of Animal Breeding, Faculty of Veterinary Medicine, Kırıkkale University, Yahşihan, Kırıkkale, Turkey; 3 Department of Chemistry and Chemical Processing Technologies, Kırıkkale Vocational Training School, Kırıkkale University, Yahşihan, Kırıkkale, Turkey; 4 Department of Chemistry, Faculty of Art and Sciences, Kırıkkale University, Yahşihan, Kırıkkale, Turkey; 5 Department of Food Technology, Suşehri Vocational Training School, Cumhuriyet University, Suşehri, Sivas, Turkey

**Keywords:** *Capoeta tinca*, Çamlıgöze dam lake, Heavy metal, Sivas

## Abstract

The concentrations of heavy metals (Ag, Cd, Co, Cu, Ni, Pb, Zn) were analyzed in muscle, skin and liver of Anatolian Khramulya, *Capoeta tinca* (Heckel, 1843) from Çamlıgöze Dam Lake located at Central Anatolian region of Turkey. The heavy metal analysis of samples was carried out by using a flame atomic absorption spectrophotometer. Ag, Cd, Co, Pb and Zn were found in all of the examined tissues. Cu and Ni were not determined in all tissues studied. The mean concentrations of heavy metals in all of the examined tissues of *Capoeta tinca* were as follows: Ag: 0.057 ± 0.038–0.120 ± 0.051, Cd: 0.020 ± 0.004–1.451 ± 0.879, Co: 0.127 ± 0.067–0.205 ± 0.086, Pb: 1.939 ± 0.477–2.604 ± 0.393 and Zn: 0.056 ± 0.014–0.530 ± 0.129 μg/g in Çamlıgöze Dam Lake. According to international criterias and Turkish regulation, heavy metal concentrations especially Cd and Pb in Çamlıgöze Dam Lake were found above the permissible levels for examined tissues of *Capoeta tinca*. Furthermore, frequent consumption of contaminated fish is able to offer a serious public health risk. Therefore, the concentrations of metals accumulated in the fish, which are commonly consumed by public, should be monitored periodically in Çamlıgöze Dam Lake.

## Introduction

Heavy metals are natural trace components of the aquatic environment, but their levels have increased due to industrial wastes, geochemical structure, agricultural and mining activities. All these sources of pollution affect the physiochemical characteristics of the water, sediment and biological components, and thus the quality and quantity of fish stocks [1-3]. In recent years, and based on the importance of fish as a part of a healthy diet, there has been a notable promotion of fish consumption. Fish is an important source of food for humans. The nutritional benefits of fish are mainly due to the content of high-quality protein, vitamins and other essential nutrients. Moreover, unlike fatty meat products, fish are not high in saturated fat. Also it has been advised that fish should be consumed two or three times weekly, because of the pharmaceutical effects of omega three polyunsaturated fatty acids, which exist abundantly in fish oil [4-6].

Anatolian Khramulya, *Capoeta tinca* (Heckel, 1843) is a species of the family Cyprinidae and has a wide distribution in western Asia. *Capoeta tinca* has a wide distribution in North and Northwest Anatolia of Turkey and lives in systems that are hydrologically connected to the Black Sea. *Capoeta tinca* can adapt very easily to changes in water regime, it occurs both in lotic and lentic habitats so this species has economic value as a commercial fish from natural and man-made lakes. Becuase of its delicious flesh, people perfer this fish species to consume as food and it is so important commercially in Turkey [7-12]. To date, heavy metals in *Capoeta tinca* from Çamlıgöze Dam Lake has not been directly studied. For Çamlıgöze Dam Lake, heavy metals in *Capoeta tinca* are given for the first time. The aim of this study was carried out to investigate accumulation of heavy metals (Ag, Cd, Co, Cu, Ni, Pb, Zn) in muscle, skin and liver tissues of Anatolian Khramulya, *Capoeta tinca* (Heckel, 1843) from Çamlıgöze Dam Lake located at Central Anatolian region of Turkey.

## Materials and methods

### Study area

**Figure 1:**
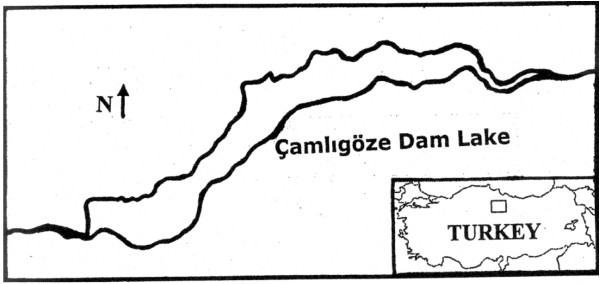
Çamlıgöze Dam Lake.

Çamlıgöze Dam Lake is located at Central Anatolian region of Turkey (Figure 1). Geographical cordinates of Çamlıgöze Dam Lake are 40° 13' 45" N, 38º 04' 36" E). The province of Sivas is located at the eastern part of the Central Anatolian region of Turkey. Çamlıgöze Dam Lake is situated approximately 140 km north-east of Sivas province centre. The Çamlıgöze Dam was constructed between 1987 and 1998 on the Kelkit Stream, a tributary of Yeşilırmak River. Çamlıgöze Dam is a 37 m high rockfill a power plant. The water of Çamlıgöze Dam Lake is mainly used for produce electrical energy, aquaculture, commercial fishing, irrigation, and recreation. The surface area and maximum depth of the Çamlıgöze Dam Lake are 5 km^2^ and 30 m respectively. Average capacity of Çamlıgöze Dam Hydroelectric Station is 102 GWh/year [13,14].

### Sampling and sample preparation

Fish samples were caught with nets of various mesh sizes (40–60 mm) between 11 January 2011 and 28 March 2011. Eleven specimens of *Capoeta tinca* were caught from Çamlıgöze Dam Lake. Fish samples were transferred to the laboratory to record age, sex, total body length and total wet weight. Body weight and total length of each individuals were measured with a precision of 0.1 g and 0.1 cm. Fish samples were washed with water, packed in polyethylene bags and stored at −20°C until analysis. Scales taking from under dorsal fin were used for age determination. For this purpose, scales were kept in 3% NaOH solution for 24 hours, and than washed in distilled water and treated with 70% alcohol solution [15]. After cleaning the scales were examined under a steromicroscope to allow age for determination. Sexual characteristics in this species were determined on gonadal tissues naked eye or using a microscope. Males differ from females morphologically by the presence of breeding tubercules formed on the head during the spawning period. Approximatelly 1 or 2 g of the muscle, skin and liver samples were dissected from 11 fish specimens. Soft tissues were extracted from each fish samples using a plastic knife. After they were individually transferred to 20 mL glass vials previously washed with 0.1 N nitric acid, dried, and weighed, they were dried in an oven for 24 hours at 105°C and kept in an oven. Then samples were kept at room temperature for 24 hours by adding 3 mL nitric acid. Then samples can withstand heat very low heats on a hot metal plate have been mineralized until the color disappears slowly heated vapors. Then the samples were added to 1 mL sulfuric acid. The samples were added into the 1–2 drops of nitric acid. The digested samples were diluted to 50 mL with distilled water [16,17]. The solution was transferred and filtered through 0.45 μm nitrocellulose membrane filter and ready for analysis [18].

Standard solutions for calibration graphs were prepared from stock solutions. All chemicals used for experiments and analyses were of analytical grades. Stock solutions of 1000 mg/L Cu (II), Ag (I), Co (II), Ni (II), Cd (II), Pb (II) and Zn (II) were supplied by Merck Company and used without further purification. Standard solutions for each metal ion were prepared from stock solutions in 25 mL flasks. Calibration graph for each batch of experiments were re-constructed by using the standard solutions. Analysis of the heavy metals in the fish samples were carried out using a flame atomic absorption spectrophotometer (GBC model 932AA) to determine the concentrations of cadmium (Cd), cobalt (Co), copper (Cu), lead (Pb), nickel (Ni), silver (Ag) and zinc (Zn). The concentrations of heavy metals were expressed as μg/g (wet weight). The absorption wavelength were as follows: 328.100 nm for Ag; 228.802 nm for Cd; 228.616 nm for Co; 327.393 nm for Cu; 231.604 nm for Ni; 217.000 nm for Pb and 213.900 nm for Zn. The detection limits were 0.1–11 μg/g for Ag; 0.2–1.8 μg/g for Cd; 0.1–9 μg/g for Co; 1–5 μg/g for Cu; 0.2–0.8 μg/g for Ni; 1.5–20 μg/g for Pb and 0.2–1.5 μg/g for Zn.

### Statistical analysis

One way analysis of variance was performed with Minitab 13.0 statistical package program on the data obtained in this study. Important differences in the mean values were tested with Duncan’s multiple range test.

## Results

A total of eleven specimens of Anatolian Khramulya, *Capoeta tinca* were caught from study area. The specimens were examined consisted 10 females and 1 male. The ages of the captured specimens of Anatolian Khramulya (*Capoeta tinca*) ranged from 5 to 9 years and the 8rd group was dominant in the population. The total length of the Anatolian Khramulya population in Çamlıgöze Dam Lake ranged from 31.3 cm to 47.7 cm. Weights of the Anatolian Khramulya ranged between 405.2 g and 1335.7 g. The mean total length of all Anatolian Khramulya samples was determined 41.081 ± 1.329 cm. The mean weight of all Anatolian Khramulya samples was determined 966.463 ± 82.867 g in Çamlıgöze Dam Lake. Some morphometric and biological characteristics of fish samples (minimum, maximum, mean and standard error) are given in Table 1.

**Table 1:** **Some morphometric and biological characteristics of*Capoeta tinca***

**Species**	**N**	**F/M**	**Age**	**Total length (cm)**	**Weight (g)**
			**Min.–Max.**	**Min.–Max.**	**Min.–Max.**
			**Mean ± SE**	**Mean ± SE**	**Mean ± SE**
			5–9	31.3–47.7	405.2–1335.7
*Capoeta tinca*	11	10♀/1♂	7.636 ± 0.387	41.081 ± 1.329	966.463 ± 82.867

The mean concentrations of heavy metals in muscle, skin and liver of Anatolian Khramulya, *Capoeta tinca* are summarized in Table 2. Ag, Cd, Co, Pb and Zn were detected in all of the examined tissues. Cu and Ni were not detected in all of the examined tissues. The mean heavy metal concentrations were determined in muscle as follows; Ag: 0.120 ± 0.051 μg/g, Cd: 0.218 ±0.203 μg/g, Co: 0.135 ± 0.073 μg/g, Pb: 2.604 ± 0.393 μg/g and Zn: 0.056 ± 0.014 μg/g. The mean heavy metal concentrations were determined in skin as follows; Ag: 0.111 ± 0.047 μg/g, Cd: 0.020 ± 0.004 μg/g, Co: 0.205 ± 0.086 μg/g, Pb: 2.373 ± 0.373 μg/g and Zn: 0.530 ± 0.129 μg/g. The mean heavy metal concentrations were determined in liver as follows; Ag: 0.057 ± 0.038 μg/g, Cd: 1.451 ± 0.879 μg/g, Co: 0.127 ± 0.067 μg/g, Pb: 1.939 ± 0.477 μg/g and Zn: 0.289 ± 0.036 μg/g. The highest concentrations were found in muscle (Pb: 2.604 ± 0.393 μg/g and Cd: 0.218 ±0.203 μg/g), in skin (Pb: 2.373 ± 0.373 μg/g and Zn: 0.530 ± 0.129 μg/g) and in liver (Pb: 1.939 ± 0.477 μg/g and Cd: 1.451 ± 0.879 μg/g) of *Capoeta tinca* from Çamlıgöze Dam Lake (Table 2). Mean concentrations in the muscle, skin and liver of *Capoeta tinca* were found as follows: Pb > Cd > Co > Zn > Ag; Pb > Zn > Co > Ag > Cd; Pb > Cd > Zn > Co > Ag. The distribution patterns of Ag and Pb in tissues of *Capoeta tinca* follows the order: muscle *>* skin *>* liver; Cd follows the order: liver > muscle > skin; Co follows the order: skin > muscle > liver and Zn follows order: skin > liver > muscle.

**Table 2:** **Mean heavy metals concentrations in different tissues of*Capoeta tinca*(μg/g)**

	**Ag**	**Cd**	**Co**	**Pb**	**Zn**
**Tissues**	**Mean ± SE**	**Mean ± SE**	**Mean ± SE**	**Mean ± SE**	**Mean ± SE**
**Muscle**	0.120 ± 0.051	0.218 ± 0.203	0.135 ± 0.073	2.604 ± 0.393	0.056 ± 0.014c
**Skin**	0.111 ± 0.047	0.020 ± 0.004	0.205 ± 0.086	2.373 ± 0.373	0.530 ± 0.129a
**Liver**	0.057 ± 0.038	1.451 ± 0.879	0.127 ± 0.067	1.939 ± 0.477	0.289 ± 0.036b
**P**	0.577	0.127	0.730	0.527	0.001*

The asteriks notation (*: P < 0.001; P > 0.05) shows the significance level in the Table.

The mean concentration of Ag in muscle, skin and liver, was 0.120 ± 0.051; 0.111 ± 0.047; and 0.057 ± 0.038 μg/g. The data showed that, muscle accumulated the highest concentration while liver accumulated the lowest. The mean Cd concentrations were 0.218 ± 0.203 μg/g in muscle, 0.020 ± 0.004 μg/g in skin and 1.451 ± 0.879 μg/g in liver. The data revealed that, liver accumulated the highest concentration of Cd while skin accumulated the lowest concentration. The mean Co concentrations were 0.135 ± 0.073 μg/g in muscle, 0.205 ± 0.086 μg/g in skin and 0.127 ± 0.067 μg/g in liver. The data revealed that, skin accumulated the highest concentration of Co while liver accumulated the lowest concentration. The mean Pb concentrations were 2.604 ± 0.393 μg/g in muscle, 2.373 ± 0.373 μg/g in skin and 1.939 ± 0.477 μg/g in liver. The data revealed that, muscle accumulated the highest concentration of Pb while liver accumulated the lowest concentration. The mean Zn concentrations were 0.056 ± 0.014 μg/g in muscle, 0.530 ± 0.129 μg/g in skin and 0.289 ± 0.036 μg/g in liver. The data revealed that, skin accumulated the highest concentration of Zn while muscle accumulated the lowest concentration (Table 2).

The significance levels of heavy metal accumulation in the tissues were defined with Duncan’s multiple range test. Letters a, b and c showed differences among tissues. There are important statistical differences, especially at the level of Zn accumulation in tissues (P < 0.001). The highest accumulation of Zn was observed in skin (with 0.530 ± 0.427 μg/g) while the lowest accumulation of Zn was observed in muscle (with 0.056 ± 0.049 μg/g). There are no statistical differences, among the concentrations of Ag, Cd, Co and Pb in tissues (P > 0.05). According to the results of analysis derived through atomic absorption spectrometry, it is confirmed that heavy metals accumulate in different tissues. The highest Ag concentration was observed in muscle of *Capoeta tinca* (0.120 ± 0.051 μg/g), while the lowest (0.057 ± 0.038 μg/g) was in liver. The highest Cd concentration was observed in muscle of *Capoeta tinca* (1.451 ± 0.879 μg/g), while the lowest (0.020 ± 0.004 μg/g) was in skin. The highest Co concentration was observed in skin of *Capoeta tinca* (0.205 ± 0.086 μg/g), while the lowest (0.127 ± 0.067 μg/g) was in liver. The highest Pb concentration was observed in liver of *Capoeta tinca* (2.604 ± 0.393 μg/g), while the lowest (1.939 ± 0.477 μg/g) was in liver. The highest Zn concentration was observed in skin of *Capoeta tinca* (0.530 ± 0.129 μg/g), while the lowest (0.056 ± 0.014 μg/g) was in muscle (Table 2).

## Discussion

There are various studies on the heavy metal levels in *Capoeta tinca* from different freshwater ecosystems in Turkey [19-24]. The mean Cd, Pb and Zn values in muscle and liver of *Capoeta tinca* obtained in this study were lower than those obtained by Ağcasulu [20] in *Capoeta tinca* from Çeltikçe Stream. Similarly, the mean Cd and Zn values in muscle of *Capoeta tinca* obtained in this study were lower than those obtained by Mendil *et al*. [19] in *Capoeta tinca* from Yeşilırmak River. The mean Zn values in all of the examined tissues of *Capoeta tinca* obtained in this study were lower than those obtained by Mendil and Uluözlü [22] in *Capoeta tinca* from Bedirkale Dam Lake and Ataköy Dam Lake. The mean Zn values in all of the examined tissues of *Capoeta tinca* obtained in this study were lower than those obtained by Mendil *et al*. [23] in *Capoeta tinca* from Yeşilırmak River. The mean Cd values in muscle and liver of *Capoeta tinca* obtained in this study were lower than those obtained by Canberk *et al*. [21] in *Capoeta tinca* from Porsuk River. The mean Cd values in muscle of *Capoeta tinca* obtained in this study were lower than those obtained by Akbulut and Tuncer [24] in *Capoeta tinca* from Kızılırmak River Basin. The mean Pb values in muscle of *Capoeta tinca* obtained in this study were higher than those obtained by Mendil *et al*. [19] in *Capoeta tinca* from Yeşilırmak River. The mean Pb values in all of the examined tissues of *Capoeta tinca* obtained in this study were higher than those obtained by Mendil and Uluözlü [22] in *Capoeta tinca* from Ataköy Dam Lake. The mean Pb values in all of the examined tissues of *Capoeta tinca* obtained in this study were lower than those obtained by Mendil and Uluözlü [22] in *Capoeta tinca* from Bedirkale Dam Lake. The mean Pb values in muscle and liver of *Capoeta tinca* obtained in this study were higher than those obtained by Canberk *et al*. [21] in *Capoeta tinca* from Porsuk River. Mean Cd concentrations ranged from 0.020 ± 0.004 μg/g to 1.451 ± 0.879 μg/g in all of the examined tissues of *Capoeta tinca* in Çamlıgöze Dam Lake. These values are similar to those reported previously [19,23].

A number of metal ions are essential for biological systems as Na, K, Ca, Mn, Fe, Co, Cu, Mo and Zn. Small quantities of B, Si, V, As, Se, Ni, Nb, Rb, Sr, and Ti were found essential for living organism. Some other metals are non essential such as Cd, Pb, Hg [25,26]. The heavy metals are the most important forms of pollution and they may accumulate in the tissues of fish which are often at the top of the aquatic food chain. They are accumulated in human tissues and may be the cause of some diseases fish may concentrate large amounts of metals from the water and they might be toxic for human consumption [27-30]. Among the different metals analyzed, Pb and Cd are classified as toxic metals, which causes chemical hazards and therefore maximum residual levels have been prescribed for human consumption by various agencies of food standards [31-33]. Pb and Cd are biologically non essential metals which are acumulated in human tissues and harmful to human health [34].

The permissible limits proposed by the FAO, WHO and Turkish legislation established the following maximum levels for the metals studied, above which consumption is not permitted: 0.1 μg/g for Cd, 5 μg/g for Cu, 50 μg/g for Zn and 0.5 μg/g or 1 μg/g for Pb [35-37]. According to international criterias and Turkish regulation, heavy metal concentrations especially Cd and Pb in Çamlıgöze Dam Lake were found above the permissible levels for examined tissues of *Capoeta tinca*. However the concentrations of Zn (0.056 ± 0.014 μg/g in muscle; 0.530 ± 0.129 μg/g in skin; 0.289 ± 0.036 μg/g in liver) was determined lower than the acceptable limits. Cu was not detected in all of the examined tissues of *Capoeta tinca*. There is also legislation in other countries regulating the maximum concentration of metals. For example, Spanish legislation limits the levels for Cd at 1 μg/g, Cu at 20 μg/g and Pb at 2 μg/g [38,39]. According to Spanish legislation limits, heavy metal concentrations Pb in Çamlıgöze Dam Lake were a bit above the permissible levels for edible tissues of *Capoeta tinca*. Heavy metal concentration Cd was above the permissible levels in liver. A comparison with the European Community food standards [32], for fish Cd: 0.05–0.10 μg/g and Pb: 0.2–0.4 μg/g. According to European Community food standards, heavy metal concentrations Cd and Pb in Çamlıgöze Dam Lake were found above the permissible levels for examined tissues of *Capoeta tinca*.

## Conclusion

The results obtained from this study will provide information for the background levels of metals in common fish species of the lake. To the best of our knowledge, the heavy metal concentrations of Anatolian Khramulya, *Capoeta tinca* are presented in Çamlıgöze Dam Lake for the first time in this study. Our results showed that the concentrations of Cd and Pb detected in tissues of Anatolian Khramulya, *Capoeta tinca* from Çamlıgöze Dam Lake were found above the FAO, WHO, ITS and EC maximum permissible limits.

## Competing interests

The authors declare that no conflict of interest.

## Authors’ contributions

All authors contributed to the manuscript. All persons listed as authors have read, contributed to preparing the manuscript and attest to the validity and legitimacy of the data and its interpretation, and agree to its submission to “Iranian Journal of Environmental Health Science & Engineering”. No person more than the authors listed have contributed significantly to its preparation. All authors read and approved the final manuscript.
